# Unsupervised Clustering of 41,728 Emergency Department Visits: Insights into Patient Profiles and KTAS Reliability

**DOI:** 10.3390/healthcare13233073

**Published:** 2025-11-26

**Authors:** Jongsun Kim, EunChul Jang, SoonChan Kwon, MyoungJe Song

**Affiliations:** 1 Department of Emergency Medicine, Catholic Kwandong University, International St. Mary’s Hospital, Incheon 22711, Republic of Korea; pucomania@gmail.com; 2 Department of Occupational and Environmental Medicine, Soonchunhyang University Cheonan Hospital, Cheonan 31151, Republic of Korea; oemdr10@gmail.com (E.J.); 91ksc@hanmail.net (S.K.)

**Keywords:** Korean Triage and Acuity Scale (KTAS), unsupervised learning, cluster analysis triage, emergency medicine

## Abstract

**Introduction:** In the emergency room, it is essential to quickly and accurately classify the patients’ various severities. However, existing five-stage classification systems, such as the Korean Emergency Patient Classification Tool (KTAS), do not sufficiently reflect the physiological and clinical heterogeneity of all patients, so there is a possibility of under-classification in some age groups or specific symptom groups. **Methods:** A retrospective cross-sectional study was conducted using KTAS and the physiological and clinical data of 41,728 patients who visited the emergency room of a university hospital in Incheon in 2022. K-prototypes unsupervised cluster analysis incorporating demographic, physiological, and clinical variables was applied, and the number of clusters was determined as the optimal value through the Silhouette, Dunn, and Davies–Bouldin indicators. Dimension reduction was performed by UMAP, and differences between clusters were compared by *t*-test, Mann–Whitney U, and chi-square test. **Results:** Two different clusters were identified. Cluster 0 was a stable patient group with a mean age of 58 years and an average arterial pressure of 104 mmHg. On the other hand, Cluster 1 was a young but physiologically unstable patient group with an average age of 46 years and an average arterial pressure of 90 mmHg. There were significant differences in age, MAP, heart rate, respiratory rate, body temperature, and pain scores between clusters (*p* < 0.001), and a moderate association was observed between KTAS classification and clusters (Cramer’s V = 0.208). **Discussion:** This study suggested the possibility of early identification of high-risk groups in the emergency room and efficient resource allocation by identifying potential patient heterogeneity that KTAS cannot detect through unsupervised learning. This approach can be used as a basis for precision triage and patient-centered emergency medical policy establishment by supplementing rather than replacing the existing classification system.

## 1. Introduction

An emergency department (ED) is a space where patients of various severity visit at the same time, and efficient patient classification is a key factor in patient safety and medical resource allocation [1,2]. Although widely used five-stage triage tools (e.g., KTAS, ESI, CTAS, and MTS) in several countries, including South Korea, have been proven to be clinically valid and reliable, the risk of under-classification and over-classification in certain age or symptomatic groups is still reported [3,4]. In particular, the consistency and sensitivity of classification are observed to decrease in the elderly and pediatric patient groups, which can directly affect the safety of patients [5,6].

Recent studies have explored data-driven approaches to supplement the limitations of these traditional triage systems. Machine learning and natural language processing-based models can assist clinicians’ subjective judgment to improve triage accuracy [7], while models that integrate vital signs and Early Warning Scores have been reported to reflect patient acuity more precisely [1]. Furthermore, beyond simple classification, there is active research on developing risk scores that can predict the likelihood of hospital admission using only the information available at the triage stage [2].

Meanwhile, there has been a recent increase in large-scale electronic medical records (EMR)-based studies to identify latent subgroups within patient populations using unsupervised learning techniques such as clustering. Studies have been reported to assess the efficiency of healthcare resource allocation by classifying detailed profiles of frequent users [7,8,9] using cluster analysis and deriving a patient group that reflects multiple morbidity patterns [10]. Furthermore, studies that have identified the risk of revisiting patients with drug-related problems through cluster analysis [11] and assessed ED operational efficiency through cluster operational characteristics suggest that data-driven cluster analysis can be applied not only clinically but also from an operational perspective [12].

While the reliability of the Korean Triage and Acuity Scale (KTAS) has been proven in several studies [3], variables still exist in the process of clinical application. Further studies are needed on how consistently KTAS results are applied in real-world clinical situations, as they may vary depending on the patient’s major complaints and modifiers. It is of great significance to analyze the correlation between patient characteristics and KTAS classification by applying an unsupervised learning approach to large-scale emergency room data beyond simple reliability verification of a single tool. Therefore, this study conducted a cluster analysis using the K-prototypes algorithm based on data from 41,728 patients who visited a single-center emergency department in 2022. This aims to (1) identify heterogeneous profiles of ED patients at the cluster level, (2) evaluate the KTAS distribution and characteristics within each cluster, and (3) present the clinical implications of this unsupervised learning approach.

## 2. Methods

### 2.1. Korean Triage and Acuity Scale (KTAS)

KTAS classification was performed according to the standard guidelines established by the Korean Society of Emergency Medicine and the Ministry of Health and Welfare [3]. Emergency physicians and residents on duty comprehensively evaluated the patients’ chief complaints, vital signs, and danger signs to classify them into KTAS Levels 1 through 5.

**Level 1:** Immediate life threat, requires cardiopulmonary resuscitation (CPR)

**Level 2:** Potential life threat, requires immediate care

**Level 3:** Serious but stable condition, requires prompt care

**Level 4:** Minor condition, care can be delayed

**Level 5:** Non-urgent

### 2.2. Research Design and Study Population

This study was a retrospective cross-sectional study conducted on patients who visited a single university hospital emergency department. Of the total 47,204, physiologically impossible values of 5491 (11.6%) and KTAS missing 8 cases (0.02%) were excluded, and the final 41,728 with complete records for key clinical variables and KTAS classification were included in the final analysis. The collected variables included demographic characteristics such as sex and age, as well as physiological parameters such as heart rate (HR), respiratory rate (RR), and body temperature (BT). We also collected pain scores based on the Numerical Rating Scale (NRS). Additionally, we calculated and included derived variables such as mean arterial pressure (MAP = [SBP + 2 × DBP]/3). In addition, the ‘Decision Time’ variable was calculated using the difference between the time of admission to the emergency room and the time to decide whether to be hospitalized or discharged, which represents the elapsed time until the patient’s treatment decision was made. This variable was included in the analysis as a result index reflecting clinical judgment and hospital bed rotation efficiency, apart from the emergency room stay time.

Chief compilation aligned free-technical data to fit the ICD-10 terminology system using semi-automated NLP-assisted mapping pipelines. After that, text normalization and keyword similarity filtering were applied to unify the transformation of the expression. Each item was classified into the ICD-10-chapter level, and as a result of the accuracy verification, the “Unknown” item showed a high level of classification completion, with only 0.004%.

### 2.3. Analysis Procedures

**Figure 1:**
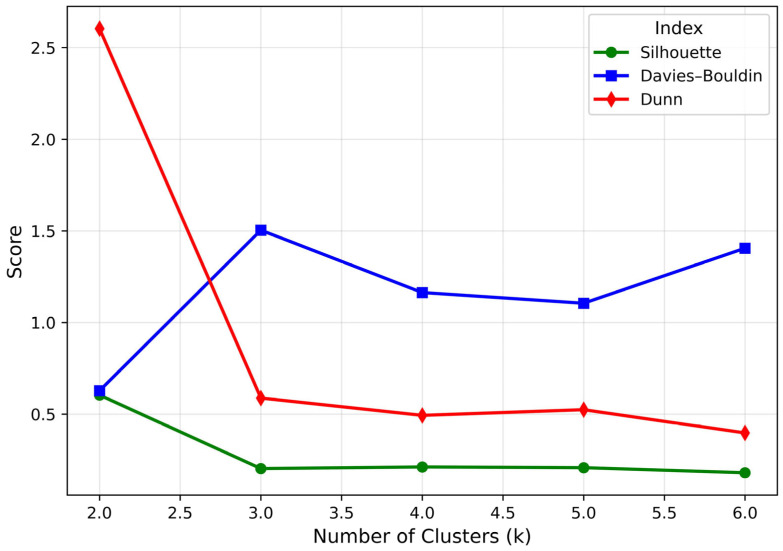
Determination of the optimal number of clusters using internal validation indices. The optimal number of clusters was calculated using three internal verification indicators (Silhouette, Dunn, and Davies–Bouldin). The Silhouette value represents the balance of cohesion and separation, and the Dunn index considers the maximum distance within a cluster compared to the minimum distance between clusters. The Davies–Bouldin index represents the average similarity between clusters, and the lower the value, the better the cluster quality. As a result of comprehensively evaluating the three indicators, it was confirmed that the balance of separation and cohesion between clusters was the best at k = 2.

Cluster analysis was performed after calculating the similarity between individuals using the Gower distance, which can consider both continuous and categorical variables at the same time. First, the merger pattern was explored through hierarchical cluster analysis, and because of comprehensively evaluating the Silhouette (0.61), Dunn (2.6), Davies–Bouldin (0.66) index, and k-prototypes cost–elbow curve, it was confirmed that the balance of cohesion and separation is the best at k = 2 (Figure 1).

**Figure 2:**
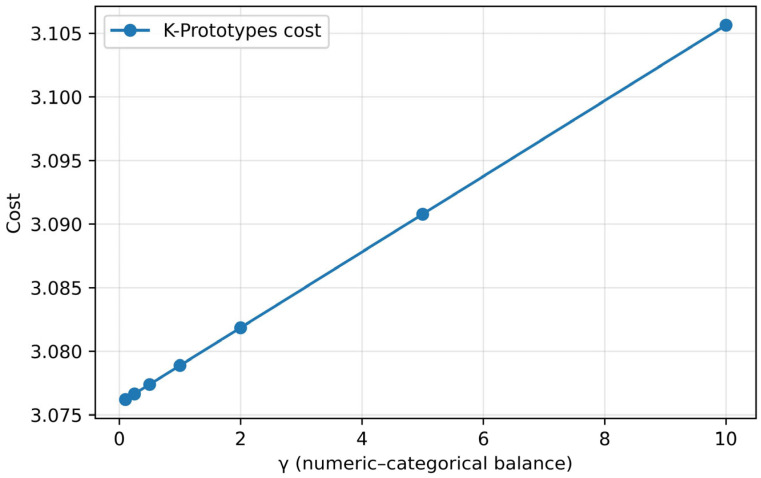
Evaluation of the γ (numeric-categorical balance) parameter in the k-prototypes algorithm. In the k-prototypes algorithm, the cost was evaluated by varying the balance weight (γ) between the continuous variable and the categorical variable in the range of 0.1–10. The interval in which cost is minimized was observed at γ = 0.1, which means that the balance between cluster cohesion and separation is the best when the contribution of the continuous variable is somewhat high. Accordingly, in this study, the γ value of the final model was set to 0.1.

To adjust the relative importance between continuous and categorical variables, the cost–elbow curve was analyzed by varying γ (continuous-categorical balance coefficient) in the range of 0.1 to 10 (Figure 2). Minimum cost was observed at γ = 0.1, and it was confirmed that increasing the weight of the categorical variable by gradually increasing the cost linearly in the subsequent section did not contribute to the improvement of cluster quality (Figure 2). The final model was set to γ = 0.1, initialization method Huang, maximum iteration of 100 times, and random seed = 42 [13]. The k-prototypes algorithm reflected the heterogeneous characteristics of emergency room data by combining k-means for continuous variables and k-modes for categorical variables, and, because of performing 100 bootstraps to verify the reproducibility of the model, the average adjusted land index (ARI) = 0.689 (95% CI 0.623–0.740) showed moderate stability. In addition, to evaluate temporal stability and external validity, the 2022 data were divided into the first and second half (1–June vs. July–December), and cluster distribution and physiological characteristics between the two time points were compared by UMAP visualization, χ^2^ test, and *t*-test.

**Figure 3:**
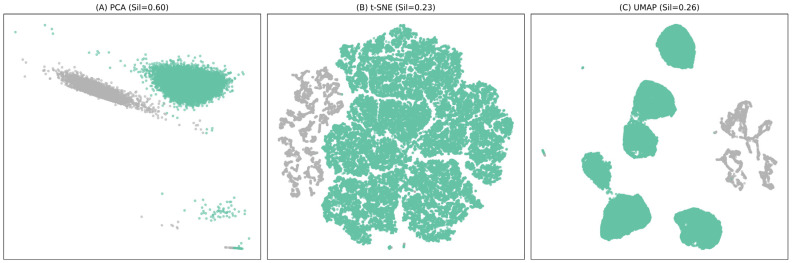
Comparison of dimensionality reduction techniques: PCA, t-SNE, and UMAP. The cluster structure was visualized in a two-dimensional space using three-dimensional reduction techniques (PCA, t-SNE, UMAP). The silhouette score of PCA (**A**) was the highest at 0.60, and the separation between clusters was the most obvious within the linear structure. t-SNE (**B**) preserved the nonlinear structure well, but the boundaries between clusters somewhat overlapped due to the high local cohesion (Silhouette = 0.23). UMAP (**C**) formed a visually distinct cluster boundary, and the silhouette coefficient (Silhouette = 0.26) was slightly lower than that of PCA at the intermediate level. As a result of a comprehensive comparison of the three techniques, it was confirmed that UMAP did not affect the maintenance of actual cluster quality (Silhouette = 0.61) while stably expressing the latent structure of nonlinear data. Cluster 0 (shown in green; Set2[0]), Cluster 1 (shown in gray; Set2[1]).

Uniform Manifold Approximation and Projection (UMAP)-based dimensionality reduction was performed to visually express the patient distribution. Parameters were set to n_n heights = 30, min_dist = 0.1, metric = ‘Euclidean’, and random_state = 42. In the UMAP results, the silhouette coefficient (coef) was 0.26 (Figure 3), showing a level of visual separation similar to that of PCA (Figure 3, Silhouette coef = 0.60) and t-SNE (Figure 3, Silhouette coef = 0.60), which is interpreted as a result of somewhat mitigating the visual separation compared to the actual cluster quality (Figure 1, Silhouette coef = 0.61) due to the characteristics of dimension reduction nonlinear mapping. Statistical analysis was performed as follows. Silhouette, Dunn, and Davies-Bouldin indicators were calculated to evaluate the cohesion and separation of clusters. Next, for the comparison of basic characteristics between clusters, an independent sample *t*-test or Mann–Whitney U test for continuous variables and a chi-square (χ^2^) test for categorical variables were used. To quantitatively evaluate the association between clusters and KTAS grades, Somers’ D, Goodman-Kruskal Gamma, and Cramer’s V coefficients were calculated, as well as the χ2 test, and an ordered logistic regression model with the final KTAS grade as a dependent variable was constructed to analyze the effects of clusters, age, and gender. The difference between clusters for decision time was compared by the Kruskal–Wallis test.

All analyses were performed in the Python ver. 3.13 environment (pandas, scikit-learn, stats models, kmodes, umap-learn), and the statistical significance level was set to *p* < 0.05.

## 3. Results

### 3.1. Characteristics of the Study Population

The final analysis for this study included 41,728 patients after excluding outliers and missing data from the total of 47,204 patients who visited an emergency department in Incheon, Republic of Korea, in 2022. The mean age was 49.7 years, with 52.1% male and 47.9% female patients.

Key vital signs were a mean HR of 85.6 beats/min, RR of 17.9 breaths/min, and BT of 36.8 °C, MAP of 95.8 mmHg, and an average NRS of 2.4 points (Table 1).

As a result of the analysis based on the ICD-10 chapter, symptoms and signs (R00–R99, 82.8%) were the most common, followed by external factors and contact factors (Z00–Z99, 8.3%), damage and addiction (S00–T98, 7.0%), musculoskeletal system (M00–M99, 1.2%), and digestive system (K00–K93, 0.7%). The “Unknown” item accounted for only 0.004% of the total.

### 3.2. Cluster Analysis

**Figure 4:**
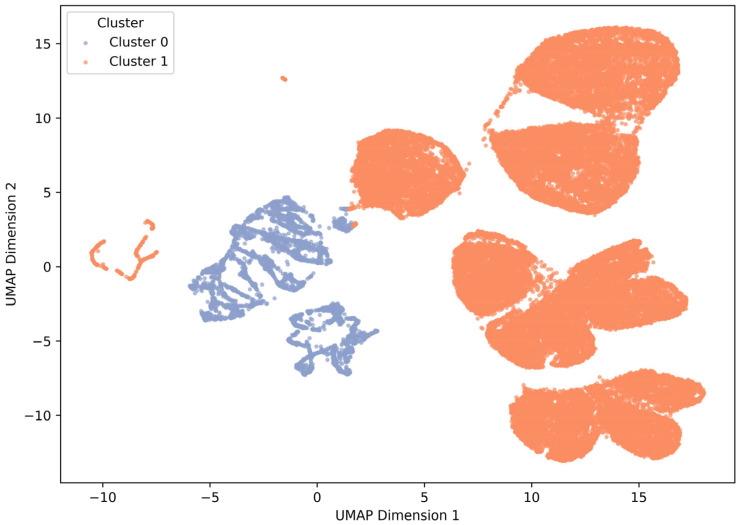
UMAP visualization of k-prototypes clustering results (k = 2). Two clusters (Cluster 0, Cluster 1) classified by the k-prototypes algorithm were visualized in a two-dimensional space through UMAP dimension reduction. Cluster 0 (blue) appeared in a relatively aggregated form and is a cluster characterized by physiological stability and high average blood pressure (MAP). On the other hand, Cluster 1 (orange) showed a wide degree of dispersion, low age, and physiologically unstable characteristics. The two clusters are clearly separated visually, reflecting differences in physiological and clinical profiles between clusters.

Cluster analysis classified the study subjects into two separate clusters using the k-prototype algorithm, with cluster 0 classified as 4465 (10.7%) and cluster 1 classified as 37.263 (89.3%) (Figure 4). Cluster 0 had an average age of 58.2 years and MAP 104.3 ± 15.7 mmHg, a stable group with a tendency to hypertension, and Cluster 1 had an average age of 45.6 years and MAP 89.7 ± 13.1 mmHg, which were young and had high physiological instability. Inter-cluster age, MAP, HR, RR, BT, and NRS showed significant differences in both the *t*-test and Mann–Whitney U test (Table 2, *p* < 0.001). As a result of the temporal stability analysis, the cluster structure was consistently maintained by period (Supplementary Figure S1). The proportion of Cluster 0 decreased from 54% in the first half to 45.6% in the second half (χ^2^ = 328.98, *p* < 0.001), but the overall phase structure and physiological characteristics of each cluster remained similar (Supplementary Table S1).

### 3.3. KTAS Distribution and Severity Patterns

**Figure 5:**
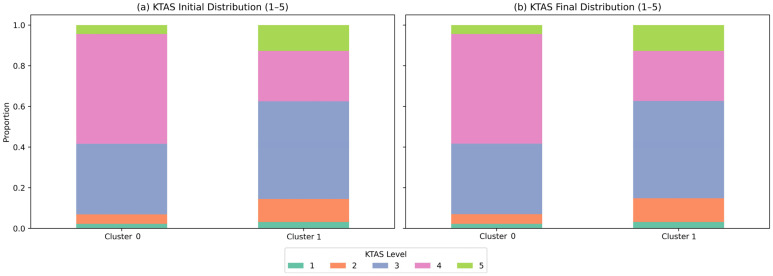
Comparison of initial and final KTAS distributions between clusters. (**a**,**b**) are cumulative ratio graphs showing the distribution of the initial and final classifications of KTAS by cluster, respectively. In Cluster 0, KTAS stages 3–4 accounted for about 86% of the total, and the proportion of relatively stable moderate patients was high. On the other hand, in Cluster 1, the proportion of patients with KTAS stage 2 or higher (moderate or higher) was relatively high, and it was identified as a cluster with a larger clinical severity (χ^2^ = 2032.18, df = 4, *p* < 0.001).

The initial and final distribution of KTAS was clearly different between the two clusters (Figure 5). In Cluster 0, the KTAS stage 3–4 accounted for 86%, whereas in Cluster 1, the proportion of patients with moderate or higher KTAS stage 2 and higher was relatively high (χ^2^ = 2032.18, df = 4, *p* < 0.001). As for the correlation strength indicators, Somers’ D = 0.001 (*p* = 0.001), Goodman–Kruskal Gamma = −0.164 (*p* < 0.001), and Cramer’s V = 0.208 (*p* < 0.001), a statistically significant but moderate association between KTAS and the cluster was confirmed. In addition, analyzing the transition matrix between the initial and final KTAS classification (Supplementary Figure S2), most patients maintained the same class (≥99%), but significant asymmetry was observed in the Stuart–Maxwell test (χ^2^ = 175.0, df = 16, *p* < 0.001). This suggests that the cluster has a clinical distinction independent of the KTAS classification, but there is a minute class shift during the classification reassessment process.

### 3.4. Sequential Logistic Regression

As a result of ordinal logistic regression analysis (Table 3), in a model with KTAS final grade (1 = most urgent, 5 = minor) as the dependent variable, the cluster variable (coef = 0.415, *p* < 0.001) showed a significant positive association with high KTAS grade (i.e., increased severity). On the other hand, age (coef = −0.022, *p* < 0.001) showed a negative direction, and KTAS grade tended to decrease (i.e., to be classified as more severe) with older age. Male sex (coef = −0.266, *p* < 0.001) was also associated with lower KTAS grade, and men were more likely to be evaluated as severe than women under the same conditions. The goodness-of-fit of the model was good with Log-likelihood = –60,310, AIC = 1.206 × 10^5^, and the age distribution difference between clusters was clear with the standardized difference (standardized difference) = −2.48. These results suggest that Cluster 1 is a relatively elderly and physiologically unstable patient group, which is a group with higher KTAS severity.

### 3.5. Decision Time Analysis

**Figure 6:**
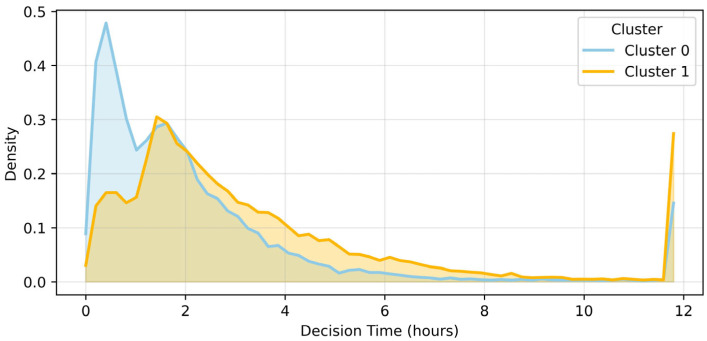
Density distribution of decision time between clusters. This is a density distribution diagram comparing the decision time (decision time) of hospitalization or discharge by cluster. Cluster 0 (blue) was determined relatively quickly with a mean of 2.12 ± 3.59 h and a median of 1.00 h (quartile range between 0.50 and 2.48). On the other hand, cluster 1 (orange) showed a tendency to delay decisions with a mean of 3.64 ± 6.89 h and a median of 2.24 h (1.36 to 3.85). The difference between the two clusters was found to be statistically significant in the Kruskal–Wallis test (H = 1323.69, *p* < 0.001).

There was a significant difference between clusters in the time (decision time) between hospitalization or exit decisions (Kruskal–Wallis H = 1323.69, *p* < 0.001). Cluster 0 averaged 2.12 ± 3.59 h (median value 1.00 [0.50–2.48]) and Cluster 1 averaged 3.64 ± 6.89 h (median value 2.24 [1.36–3.85]), indicating decision delay in Cluster 1 (Figure 6).

## 4. Discussion

This study performed unsupervised cluster analysis on over 40,000 emergency department patient records to derive patient characteristics that traditional classification systems, such as KTAS, may not capture. While KTAS is a rapid triage tool based on vital signs and symptoms, it has limitations in adequately reflecting factors such as age, chronic disease burden, and long-term prognosis [7]. Our unsupervised cluster analysis complements these limitations, demonstrating that distinct patient profiles can be distinguished even within the same KTAS level [14].

The two clusters showed clearly distinct physiological and clinical profiles. Cluster 0 was a group of stable patients with a tendency to hypertension with a mean age of 58 years and an average arterial pressure of 104 mmHg, mainly a mild disease, while Cluster 1 was identified as a younger but physiologically unstable cluster with an average age of 46 years and an average arterial pressure of 90 mmHg. Cluster 1 has a high heart rate and a high proportion of KTAS moderate patients, which is likely to be associated with acute trauma and stress response. The association between KTAS and clusters was moderate (Cramer’s V = 0.208), suggesting that KTAS does not fully reflect physiological instability. Furthermore, it shows that the decision time is significantly longer in Cluster 1 (3.64 h vs. 2.12 h, *p* < 0.001), which can act as a major factor for decision delay in the emergency room. These results suggest that the cluster-based approach complements the limitations of KTAS and may contribute to the prediction of physiological risks and optimization of resource allocation in emergency patients.

These cluster characteristics are consistent with various patient profiles suggested in previous studies. For example, clusters of elderly patients are often characterized by multiple diseases and high readmission rates [15], while traumatic patients show different prognoses depending on coagulation response and shock status [16]. Furthermore, frequent visits to the emergency room have been reported for various reasons, such as mental health issues or chronic diseases, and customized interventions are needed rather than a single approach [17]. The clusters derived in this study are also consistent with this international evidence and are significant in that they identify groups of patients at latent risk that are not accounted for by a single KTAS criterion [18].

The two clusters derived in this study represent distinct patient types that can complement the KTAS classification. Cluster 0 is a stable middle-aged and elderly group with a tendency towards high blood pressure, requiring chronic disease management and outpatient follow-up linkage. On the other hand, Cluster 1 is a relatively young but physiologically unstable patient group, with a high heart rate and pain score, and a high proportion of KTAS patients over moderate, requiring rapid treatment and real-time monitoring systems [12,17]. When these cluster characteristics are reflected, they can be used as a basis for advancing the emergency room resource relocation strategy [13,19,20,21,22,23]. For example, it is possible to expand monitoring manpower for patient groups with high physiological instability or strengthen the chronic disease management linkage system for stable patients. As a result, the unsupervised cluster approach of this study can be used as a basic tool for precision triage and data-driven risk stratification. This study showed that unsupervised cluster analysis is not simply a tool to classify emergency room patients by severity, but a sophisticated patient profiling methodology that can support clinical and administrative decision making. By applying the k-prototypes algorithm [24], which can process mixed-type data in an integrated manner, and reflecting the verification procedure suggested in a recent machine learning study in the field of emergency triages, a structural framework for data-based interpretation of patient heterogeneity in emergency medical settings was presented [25,26]. This approach has the potential to simultaneously improve the safety and efficiency of emergency patient management by supplementing existing classification systems such as KTAS.

To further generalize the results of this study, future studies plan to conduct multi-center, multi-period studies including various hospitals and periods. Through this, it is possible to verify the reproducibility and consistency of cluster structures in various environments and to suggest the possibility that the proposed unsupervised learning approach can be extended to a precision triage and risk classification system in actual clinical practice. In addition, by adding a symptom-level granularity analysis using NLP (natural language processing), it is intended to reflect the patients’ symptom information in more detail and increase the clinical interpretability of the cluster.

## 5. Conclusions

This study applied unsupervised k-prototypes cluster analysis on 41,728 emergency room patient data to identify the characteristics of potential patient groups that are not classified by the KTAS classification. As a result of the analysis, Cluster 0 was identified as a stable patient group in the middle and elderly with a tendency to hypertension, and Cluster 1 was identified as a relatively young but physiologically unstable patient group. The two clusters showed different physiological risks even within the same KTAS stage, reflecting patient heterogeneity that the existing classification system could not capture. This unsupervised cluster approach can go beyond simple severity classification and contribute to early identification of high-risk groups, optimization of resource allocation, and strategies to alleviate congestion in the emergency room. In future studies, it is necessary to expand the development of a patient-centered precision triage system by verifying the clinical progress and prognosis for each cluster using multi-center and long-term tracking data.

## Figures and Tables

**Table 1:** Baseline characteristics of study participants (*n* = 41,728).

Variable	Mean (SD)	25%	Median	75%	Max
**Age (years)**	49.7 (21.4)	32	51	65	107
**Heart rate (beats/min)**	85.6 (18.5)	73	83	97	234
**Respiratory rate (/min)**	17.9 (2.2)	16	18	18	99
**Body temperature (°C)**	36.8 (0.7)	36.4	36.7	37.0	42.0
**Mean arterial pressure (mmHg)**	95.8 (16.3)	85.7	95.0	105.3	212.7

BP: blood pressure; SD: standard deviation.

**Table 2:** Comparison of baseline characteristics between clusters.

Variable	Cluster 0 (*n* = 4465)	Cluster 1 (*n* = 37.263)	*p*-Value *
**Age (years)**	58.2 ± 14.5	45.6 ± 16.3	<0.001
**HR (/min)**	78.2 ± 12.4	90.3 ± 15.7	<0.001
**RR (/min)**	17.3 ± 2.8	19.2 ± 3.1	<0.001
**BT (°C)**	36.7 ± 0.5	37.0 ± 0.6	<0.001
**NRS**	2.1 ± 1.9	2.8 ± 2.2	<0.001
**MAP (mmHg)**	104.3 ± 15.7	89.7 ± 13.1	<0.001

HR: heart rate; RR: respiratory rate; BT: body temperature; NRS: numeric rating scale (for pain); MAP: mean arterial pressure; shock index (HR/SBP): defined as the ratio of heart rate to systolic blood pressure. * *p*-values < 0.001 for all comparisons by both *t*-test and Mann–Whitney U test.

**Table 3:** Ordered logistic regression for final KTAS levels (dependent variable).

Variable	Coefficient (β)	Std. Error	z-Value	*p*-Value	95% CI [Lower, Upper]
**Cluster (1 vs. 0)**	0.415	0.032	13.055	<0.001	[0.353, 0.477]
**Age (years)**	−0.022	0.000	−51.905	<0.001	[−0.023, −0.021]
**Sex (male = 1)**	−0.266	0.017	−15.418	<0.001	[−0.300, −0.232]
**Cutpoint 1/2**	−4.333	0.038	−113.736	<0.001	[−4.408, −4.258]
**Cutpoint 2/3**	0.523	0.014	36.194	<0.001	[0.495, 0.551]
**Cutpoint 3/4**	0.866	0.006	141.805	<0.001	[0.854, 0.878]
**Cutpoint 4/5**	0.489	0.008	59.752	<0.001	[0.473, 0.505]

Model fit: Log-Likelihood = −60,310, AIC = 1.206 × 10^5^, *n* = 47,196 Standardized difference (Age) = −2.48. As a result of ordered logistic regression with KTAS grade (1–5) as a dependent variable, cluster variables showed a significant positive relationship with high KTAS grade (increased severity), and age and male gender were associated with low severity. Cutpoint means the cumulative probability boundary value between each grade, and the fit of the model was good.

## Data Availability

The dataset analyzed in this study is derived from the electronic medical records (EMR) of the Catholic Kwandong University International St. Mary’s Hospital. Due to institutional and legal restrictions protecting patient privacy, the data cannot be shared publicly. De-identified data may be made available upon reasonable request from the corresponding author, subject to institutional review and IRB approval.

## Supplementary Materials

The following supporting information can be downloaded at: https://www.mdpi.com/article/10.3390/healthcare13233073/s1, Table S1. Temporal Stability of Cluster Characteristics (Early vs. Late 2022). Figure S1. Temporal Stability of Clustering (Early vs. Late 2022). Figure S2. Transition matrix between initial and final KTAS classifications.
