# Supplementary material for: Unsupervised Clustering of 41,728 Emergency Department Visits: Insights into Patient Profiles and KTAS Reliability
Source: Healthcare (Basel). 2025 Nov 26;13(23):3073. doi: 10.3390/healthcare13233073 (PMC12692310; doi:10.3390/healthcare13233073)
Supplement: Supplementary file 1 [file healthcare-13-03073-s001.zip › healthcare-3944653-supplementary.pdf]

Supplementary Table S1. Temporal Stability of Cluster Characteristics (Early vs. Late 2022)

Variable	Cluster	Early 2022 (Jan–Jun) Mean ± SD	Late 2022 (Jul–Dec) Mean ± SD	Δ (Change)	t/χ² (p)
**Proportion (%)**	**0**	54.0	45.6	–8.4	χ^2 = 328.98
	**1**	46.0	54.4	+8.4	(p < 0.001)
**Age (years)**	**0**	41.79 ± 17.2	46.03 ± 18.5	+4.24	t = –12.4
	**1**	49.96 ± 20.1	43.25 ± 19.8	–6.71	(p < 0.001)
**MAP (mmHg)**	**0**	86.61 ± 11.3	77.64 ± 10.8	–8.97	t = 15.8
	**1**	86.35 ± 12.0	89.07 ± 11.1	+2.72	(p < 0.001)
**Heart rate (bpm)**	**0**	88.67 ± 14.2	92.26 ± 15.4	+3.59	t = –9.4 (p < 0.001)
	**1**	88.40 ± 13.6	87.34 ± 14.8	–1.06	
**Respiratory rate (/min)**	**0**	18.08 ± 2.1	19.29 ± 2.3	+1.21	t = –8.3 (p < 0.001)
	**1**	18.70 ± 2.2	18.10 ± 2.1	–0.60	
**Body temperature (°C)**	**0**	36.85 ± 0.42	37.00 ± 0.48	+0.15	t = –6.1 (p < 0.001)
	**1**	36.66 ± 0.39	36.61 ± 0.41	–0.05	
**Pain score (NRS)**	**0**	4.36 ± 1.8	0.01 ± 0.2	–4.35	t = 21.7 (p < 0.001)
	**1**	0.02 ± 0.3	4.37 ± 1.7	+4.35	

Major physiological indicators of each cluster and the mean (±standard deviation) of pain scores were compared, and t-tests were used for continuous variables and chi-square tests were used for ratio differences.

Supplementary Figure S1. Temporal Stability of Clustering (Early vs. Late 2022)

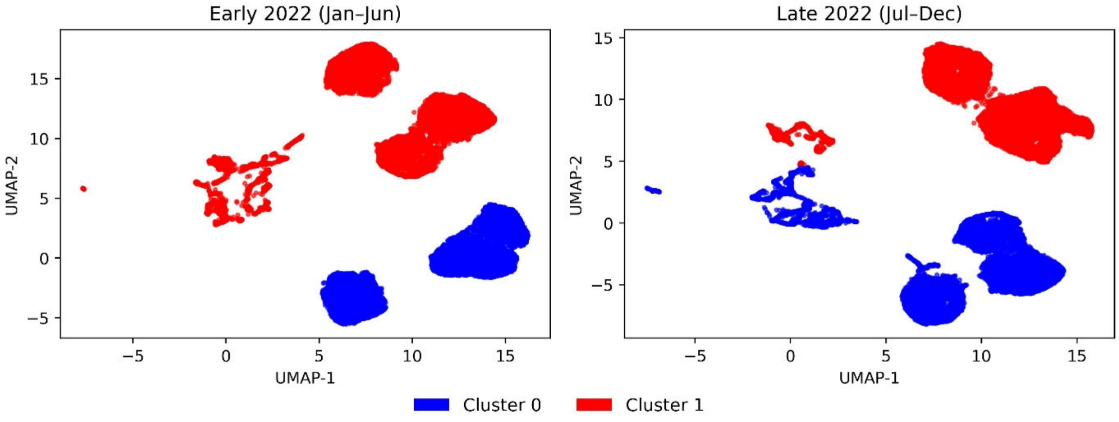

The temporal stability of the unsupervised cluster (K-means) between the first half of 2022 (January–June) and the second half (July–December). Although the proportion of the cluster changed slightly, the overall topology remained consistent, confirming the temporal reproducibility of the cluster structure.

Supplementary Figure S2. Transition matrix between initial and final KTAS classifications

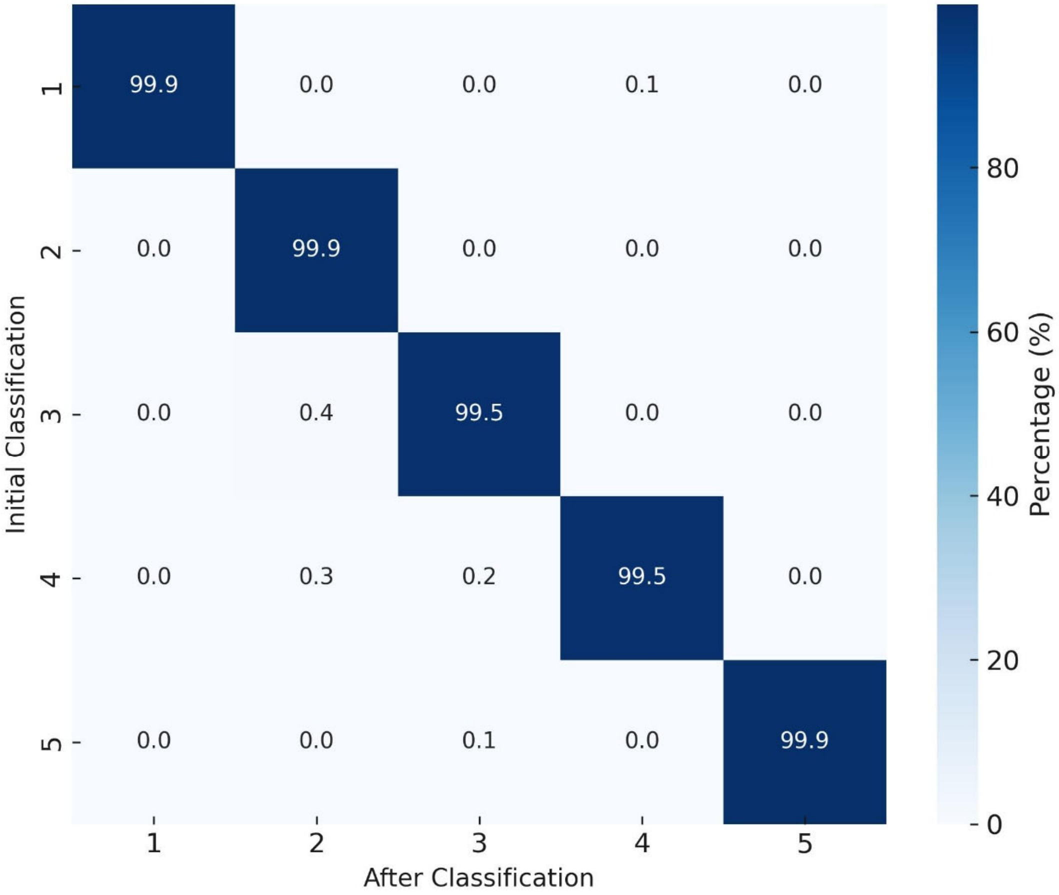

This figure shows the changes between the initial and final KTAS classification of the transition matrix form. Most patients maintained the same grade (≥ 99%), and only a fraction of the intergrade change was observed between stages 3 and 4. The Stewart-Maxwell test revealed statistically significant differences of $\chi^2 = 175.0$, df = 16, and $p < 0.001$, suggesting that there is a minute class shift in the KTAS reclassification process. These results show a consistent trend with the differences in the severity distribution among clusters presented in the body of Fig. 5.
